# Transdisciplinary research before, during and after COVID-19 vaccination in Chile: a virtuoso collaboration with future perspectives

**DOI:** 10.3389/fpubh.2024.1354645

**Published:** 2024-04-03

**Authors:** Juan Pablo Torres, Leonardo Basso, Denis Saure, Marcela Zuñiga, Andrés Couve, Mauricio Farfán, Verónica de la Maza, Nelson Campos, Miguel O’Ryan

**Affiliations:** ^1^Faculty of Medicine, Universidad de Chile, Santiago, Chile; ^2^Instituto Sistemas Complejos de Ingeniería (ISCI), Santiago, Chile; ^3^Hospital Luis Calvo Mackenna, Santiago, Chile; ^4^Faculty of Physical Sciences and Mathematics, Universidad de Chile, Santiago, Chile; ^5^Ministerio de Salud, Gobierno de Chile, Santiago, Chile

**Keywords:** COVID-19, transdisciplinary action-research, Chile, hospital bed prediction, mobile phone based mobility tracking, SARS-COV-2 IgG seroprevalence, COVID-19 vaccination

## Abstract

The COVID-19 pandemic presented numerous challenges that required immediate attention to mitigate its devastating consequences on a local and global scale. In March 2020, the Chilean government, along with health and science authorities, implemented a strategy aimed at generating relevant evidence to inform effective public health decisions. One of the key strengths of this strategy was the active involvement of the scientific community, employing transdisciplinary approaches to address critical questions and support political decision-making. The strategy promoted collaborations between the government, public and private institutions, and transdisciplinary academic groups throughout each phase of the pandemic. By focusing on pressing problems and questions, this approach formed the foundation of this report which reflects the collaborative effort throughout the pandemic of individuals from the Instituto de Sistemas Complejos de Ingeniería (ISCI), the Faculty of Medicine of the University of Chile, government authorities and industry. Early in the pandemic, it became crucial to gather evidence on how to minimize the impact of infection and disease while awaiting the availability of vaccines. This included studying the dynamics of SARS-CoV-2 infection in children, assessing the impact of quarantines on people’s mobility, implementing strategies for widespread SARS-CoV-2 polymerase chain reaction (PCR) testing, and exploring pool testing for large populations. The urgent need to reduce disease severity and transmission posed a significant challenge, as it was essential to prevent overwhelming healthcare systems. Studies were conducted to predict ICU bed requirements at the local level using mathematical models. Additionally, novel approaches, such as using cellphone mobility-based technology to actively identify infected individuals, and to optimize population sampling, were explored following the first wave of the pandemic. Chile took early action in addressing vaccination through a high-level scientific board, before vaccines became available. Studies conducted during this period included population-based immunologic evaluations of different vaccines, which helped build confidence in the population and supported the need for booster doses and potential vaccination of children. These studies and collaborations, which will be discussed here, have provided valuable insights and will inform future approaches in a post-pandemic world. Importantly, highly conservative estimates indicate that 3,000 lives and more than 300 million USD were saved by this academic-public-private collaborative effort.

## Introduction

COVID-19 has had a profound impact on public health, making it one of the most significant disasters in recent history. While the extent of its impact varied among different populations, the global excess mortality rate reached 120.3 deaths per 100,000 people, with 21 countries experiencing rates exceeding 300 deaths per 100,000 population (1). Chile, a middle-high-income country in South America with a population of 19.5 million, faced the pandemic in March 2020, seventeen months after a severe social crisis that resulted in civil unrest. The government, a conservative coalition at the time, had relatively low popular support and a highly fragmented parliament. This complex political landscape posed challenges in dealing with the pandemic amidst significant uncertainties. Bringing together a divided society and political leadership to unite efforts, dialog, sacrifices, and agree on national strategies seemed in this scenario, of extreme difficulty. However, objective assessments indicate that Chile performed relatively well, despite the tragic loss of approximately 61.3 thousand lives (2). This relative success can be attributed to the capacity to advance coordinated public health measures, active intersectoral collaborations, and research. The active engagement of the scientific community, employing transdisciplinary approaches to address critical questions and support political decisions, was a key strength of Chile’s strategy.

**Figure 1:**
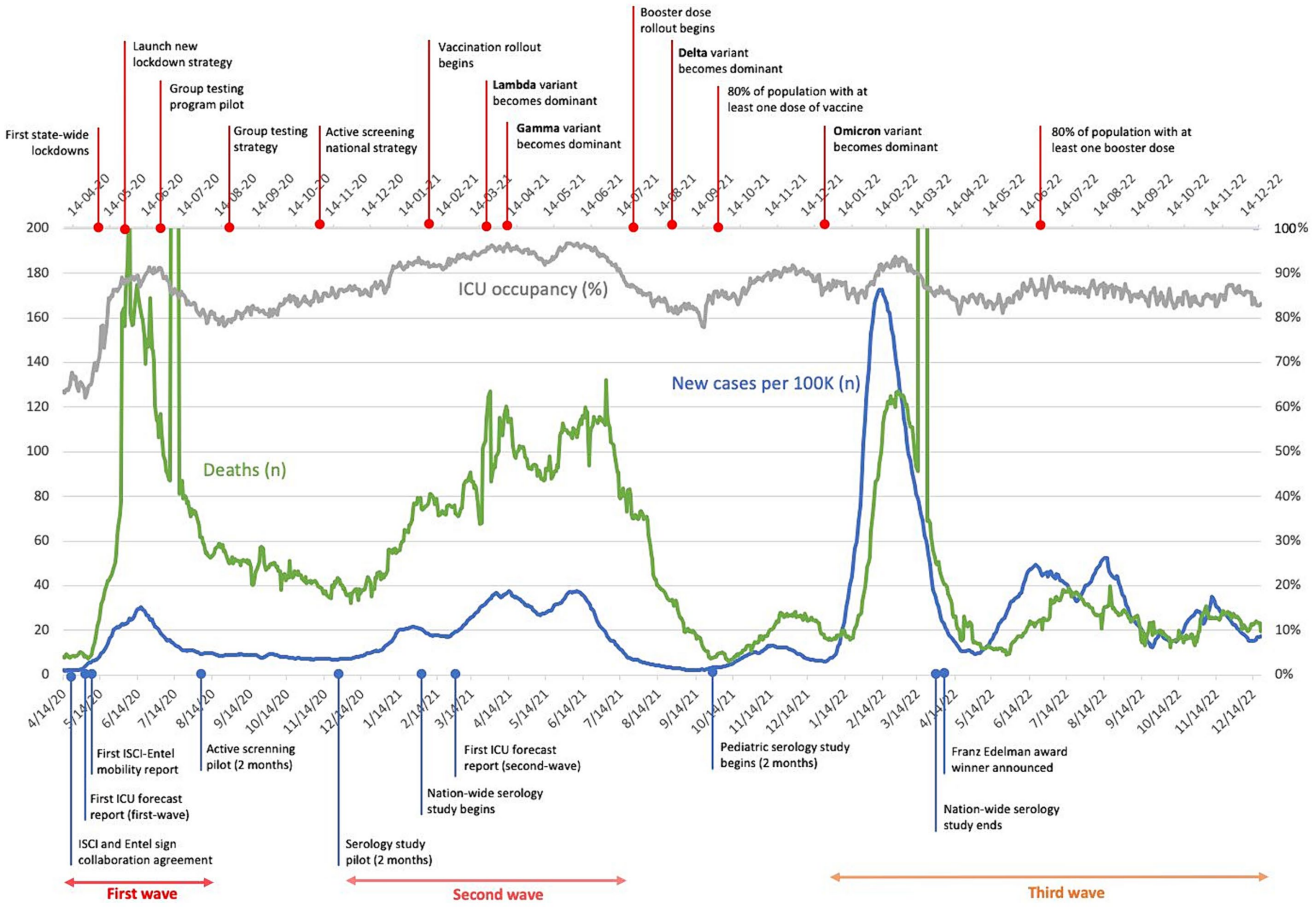
Timeline of the pandemic in Chile from 4/20 to 12/22. The magnitude of relevant pandemic outcomes are included. Milestones associated with the pandemic in general, and the project in specific are indicated in the upper and bottom axis, respectively.

Figure 1 presents the course of the pandemic in Chile, along with a timeline highlighting key public health measures implemented and the critical questions that required evidence-based responses. The first wave, from April to August 2020, witnessed a rapid increase in cases, with daily counts surpassing 5,000. This surge in cases led to a threefold increase in the demand for ICU beds and the highest daily death toll of the entire pandemic. After a decline in cases during the spring, the second wave occurred from December 2020 to July 2021, characterized by a significant increase in ICU bed requirements and deaths, although not reaching the peak levels of the first wave. This second wave was largely driven by the Gamma variant, which affected Latin America. The third wave, following another decline in cases, saw a massive increase in daily detections, surpassing 33,000 in the summer of 2022, followed by an extended wave in late autumn and early spring due to the Omicron variant. Although this wave resulted in a high overall death toll, the proportion of deaths to overall cases was lower compared to the first and second waves.

Throughout the pandemic, Chile implemented a series of public health measures based on international and internal recommendations from expert groups convened by the government, including the Presidency, Ministry of Health, and Ministry of Science and Technology. These measures aimed to contain virus entry and dissemination during the first wave, followed by mitigation strategies. The healthcare system’s emergency preparedness played a crucial role in managing the significant increase in severe patients and expanding SARS-CoV-2 diagnostic capacity nationwide. As the first wave subsided, efforts focused on increasing the detection of asymptomatic and mildly symptomatic cases and preparing for vaccination while sustaining mitigation measures and addressing healthcare needs.

The uncertain and fearful environment created by the pandemic necessitated the support and commitment of the scientific and academic community. Numerous questions emerged daily in Chile and worldwide, requiring evidence to provide valid answers. However, the political and public health authorities lacked the resources to address these questions. The scientific and academic community had to overcome their own fears and dedicate time and effort to raise relevant questions, provide evidence, and offer answers. Figure 1 presents some of the questions posed during this period, particularly those addressed by one of the main transdisciplinary collaborations described in this review. The Ministry of Science and Technology played a crucial role in fostering a network of researchers, providing resources for research initiatives, establishing an open database for information sharing, and acting as a liaison between the scientific community and the government. In this report we aim to share the collaborative efforts, throughout the pandemic, among individuals from the Instituto de Sistemas Complejos de Ingeniería (ISCI), the Faculty of Medicine of the University of Chile, government authorities and industry to address emerging questions.

The first month of the pandemic in Chile was marked by a school outbreak, which raised questions about the role of adults versus children in SARS-CoV-2 transmission. While experience with other respiratory viruses suggested that children could be significant vectors for transmission, data from the northern hemisphere indicated that children were less affected by this virus. Researchers from the Faculty of Medicine at the University of Chile addressed this question as early as April 2020 (3). Another important measure to limit virus spread was the selective implementation of quarantines, aiming to restrict movement in areas with a significant increase in cases without completely shutting down large cities. However, the effectiveness of this strategy in limiting people’s movement was unclear. To address this, a large epidemiological and technological project was initiated to track the movement of people using anonymized cell phone mobility data (4). This project compared diurnal and nocturnal tracking to identify areas where people were likely to carry asymptomatic infections. The rapid increase in cases, including severe cases, necessitated enough intensive care unit beds nationwide. To address this, an unprecedented collaboration between engineers from the Institute Sistemas Complejos de Ingeniería (ISCI) and the Ministry of Health resulted in the development of a model that predicted regional bed requirements within a two-week interval, helping to avoid the “dilemma of the last bed” scenario (5).

After the intense first winter wave, cases started to decline in spring, allowing for the implementation of new initiatives for the upcoming months. The government-sponsored initiative to convert university, public, and private laboratories into diagnostic labs using PCR technology significantly increased SARS-CoV-2 polymerase chain reaction (PCR) testing capacity. However, this was deemed insufficient, especially for active case tracing and testing of symptomatic and asymptomatic individuals to limit the spread and delay future waves. To improve testing capacity, researchers developed and validated for use in Chile “pool testing” techniques for SARS-COV-2, which were subsequently applied in specific settings such as nursing homes and eventually nationwide (6, 7). Additionally, cell phone mobility technology was utilized to identify “hotspots” within large cities, characterized by high circulation of people likely to carry asymptomatic infections. In these hotspots, the government established voluntary testing sites for asymptomatic or mildly symptomatic individuals.

Vaccine preparedness became a cornerstone of Chile’s strategy. The government established a vaccine advisory board to review all vaccine candidates and their developments. One important aspect was the initiation of vaccine trials in Chile with different manufacturers. As early as January 2021, it became evident that information on vaccine effectiveness was crucial, especially for the widely used inactivated vaccine from the Chinese laboratory Sinovac. Researchers associated with the Health Ministry conducted a countrywide effectiveness study (8), while a parallel study on immunogenicity was carried out by the ISCI and the University of Chile (9). Cell phone mobility technology was once again employed to select sites for rapid SARS-CoV-2 antibody testing, representative of regional populations. These studies revealed waning immunity after two doses of the inactivated vaccine, highlighting the need for a booster dose. Further testing demonstrated the significant benefit of a heterologous booster dose in individuals who received the inactivated vaccine as their primary dose (10, 11). Studies conducted in children, who were less affected by SARS-CoV-2 but still susceptible to infection, showed a similar response to vaccination as observed in adults (12).

In summary, collaborative transdisciplinary research in Chile played a crucial role in improving health outcomes during the COVID-19 pandemic, despite the uncertainties faced. The scientific and academic community actively engaged in addressing critical questions and supporting the national strategy. This approach gained international recognition, as evidenced by the receipt of the 2022 Franz Edelman Award for Achievement in Advanced Analytics, Operations Research, and Management Science.

The details of each research strategy are further explored below.

## The first months: urgent need for evidence and tools to understand infection dynamics

Prior to the COVID-19 crisis, Chile benefited from its geographical barriers, which provided natural insulation against certain external pathogens and pandemics like the Zika outbreak in 2014, which caused significant damage in other parts of Latin America. However, this isolation did not prove sufficient in containing global infectious agents such as H1N1 in 2009, which resulted in 368,118 confirmed cases (13). Awareness of the COVID-19 situation began in Chile through information received from China and the rest of the world, leading to strategic meetings between the Chilean government and foreign officials and experts. Local preparations for the incoming disease started as early as January 28, 2020, with the examination of incoming travelers from Chinese territory (14). Measures were implemented in airports on February 17 for symptom evaluation, followed by sanitary customs measures later in the month. Former President Sebastián Piñera^1^ addressed the nation on March 3, discussing the measures in place since January. On the same day, the first COVID-19 case within Chilean borders was confirmed in Talca, a city south of the capital, Santiago (15). Following the World Health Organization’s declaration of a global pandemic on March 11, Chile closed its borders for non-nationals and permanent residents on March 18.

There are two additional historical factors that should be considered when analyzing Chile’s response to the COVID-19 emergency. First, the crisis occurred shortly after a period of social and political unrest in late 2019 and early 2020 as mentioned above. Massive protests erupted across the country in response to increased public transportation fares, resulting in widespread damage to public and private infrastructure. This led to a declaration of constitutional exemption and curfews. This social unrest remained a concern until the arrival of COVID-19, which further complicated the response to the crisis and contributed to fragmentation.

Second, Chile established its first Ministry of Science, Technology, Knowledge, and Innovation on December 18, 2018. This ministry served as a science authority at the highest executive and political level, leading to high expectations during the COVID-19 crisis. The ministry’s emerging capacities and resources were redirected to support the pandemic effort, including efforts to coordinate the research community, provide emergency research funds, implement diagnostic capacities in universities and other sectors, and generate open-access data. The ministry also worked on a strategy to access potential vaccines as early as possible.

The first reported outbreak in Chile occurred on March 12, 2020, at a private school in Santiago. This came just 9 days after the first case of SARS-COV-2 was detected in the country and 8 days into the school year. The outbreak started with the detection of two cases, one teacher and another staff member, who had contact with parents, teachers, and children in the preschool community. The entire school community was placed under quarantine on the same day. Within 25 days, 52 cases of positive SARS-COV-2 PCR were detected, with teachers and parents being more affected compared to students.

Researchers from the Faculty of Medicine at the University of Chile conducted a finger prick lateral flow anti SARS-COV-2 based study to determine the role of children in the transmission of SARS-COV-2 as potential asymptomatic transmitters. The study included a sample of 1,099 students and 235 staff members. Antibody positivity rates were 10% for students and 17% for staff. Results showed that adults played a more significant role in the outbreak, with cases primarily detected after the school closure. It is likely that students were infected by adults, either their teachers or parents (3).

This study provided valuable information on transmission dynamics within schools, helping with early identification of outbreaks, implementation of appropriate measures, and informed decision-making regarding school reopening. It highlighted the importance of prioritizing efforts to prevent new cases among adults in scenarios where community transmission levels were low.

Conducting the study posed significant challenges due to strict quarantines in place at the time. Strategic partnerships with private companies and donors were formed to finance the processes, and active community participation was essential, with individuals self-administering the antibody tests. The success of the study can be attributed to the cohesive and experienced research team capable of designing, presenting, and executing the project efficiently. Innovative methods and contact-free epidemiological tools were implemented, which can serve as a reference for other researchers in similar situations or remote areas during the pandemic.

## The first winter surge: mitigating pandemic impact while waiting for vaccine developments

### Short-term forecasts of ICU bed requirements

By early May 2020, the rate of COVID-19 infections began to rapidly increase, posing a threat to the capacity of healthcare services to handle all incoming cases, especially the more severe ones. In mid-May, the Chilean Society of Intensive Medicine (SOCHIMI) reported that ICU beds in the capital city of Santiago, where the majority of cases were concentrated, were occupied at a worrisome rate of over 95%. As a result, planning for ICU capacity became a top priority. On May 12th, the ISCI was requested to provide short-term forecasts of ICU bed occupancy rates for regions with the highest utilization rates. Within 24 h, the first report was submitted, and subsequent reports were prepared every 2 days for several weeks before reducing the frequency to every 4 days. These reports were sent directly to the authorities, particularly the coronavirus response committee, which included the country’s President, as well as to SOCHIMI.

The ISCI team devised a solution for generating predictions of the number of ICU beds that would be needed by COVID-19 patients in every region of the country, with a 14-day time horizon. The methodology relied on utilizing an ensemble of various forecasting models, each capturing different aspects of the outbreak’s progression. The first model employed a compartmental approach, describing the flow of patients through different clinical states with a stochastic element. It considered that new patients would require an ICU bed after a specific number of days, with a given probability, and that they would be discharged after a certain number of days according to another probability distribution. However, while compartmental models have been widely used to study epidemics, they have limitations in adapting to dynamic changes in the environment. In the case of COVID-19, critical parameters such as the time delay between case identification and ICU bed requirement, the duration of mechanical ventilation needs, and the likelihood of urgent care varied over time. To address this, several autoregressive and machine learning models, capable of capturing dynamic variations, were incorporated. The forecasts generated by these different models were then combined using a trimmed mean ensemble technique.

Our approach had the ability to generate accurate forecasts, with average prediction errors of 4 and 9% for one- and two-week horizons, respectively. This outperformed other competing ensembles of forecasting models. The minister of science reported each short-term ICU forecast to the president and his crisis advisory committee, who used the forecast as input to focus efforts on adjusting the supply of ICU beds. (5)

### Mobility and the waving effect of lockdowns

The rapid spread of infection in March 2020 in the city of Santiago led to school closures and lockdowns, particularly in areas with higher case numbers. However, it was found that the effectiveness of these lockdowns varied significantly. While they were successful in reducing cases in high-income areas, middle- and low-income areas continued to experience a higher rate of infection.

To monitor the impact of lockdowns on social distancing, the ISCI team partnered with the largest telecom company in the country, ENTEL, to track the mobility patterns of the population. The team used telecommunications infrastructure data to create more detailed mobility indicators. By georeferencing devices during each connection using antenna triangulation, they obtained a representative sample of device locations. A household was assigned to each device based on nighttime connections and data was aggregated at the census zone level to maintain anonymity. Mobility patterns were identified by tracking the location of each anonymous device during morning and afternoon time-blocks, and the most frequent census zone for each device was used to assign a location for each time block. The data was then aggregated using origin–destination matrices at the census zone level.

Many of the mobility indicators were made available on an online visualization platform for the general public and health authorities. This prompt availability of mobility data allowed health authorities to respond quickly, compared to relying solely on infection rates.

The data revealed a significant disparity in mobility reductions across municipalities. Higher income areas reduced mobility by 40–50%, middle income areas by 30–40%, and lower income areas by only 20–25%. This heterogeneity in compliance with shelter-in-place mandates explained why lockdowns were less effective in controlling infections in certain areas. Furthermore, over time, the mobility indicators showed a decline in the responsiveness of mobility to shelter-in-place mandates, indicating a strong “lockdown fatigue” effect. During the second wave of lockdowns in Chile, the overall reduction in mobility averaged 18%. This diminishing effectiveness of lockdowns over time highlighted the need for alternative measures to contain the pandemic (4). Importantly, comparable results in addition to other relevant findings were reported by Mena and colleagues from the United States and the Facultad de Ciencias Biológicas, Pontificia Universidad Católica de Chile. Mena (16), and Gozzi and colleagues from Europe, United States and Universidad del Desarrollo, Chile (17).

In summary, although lockdowns initially proved effective in mitigating the spread of infections in certain regions, their impact diminished over time. The availability of mobility data provided valuable insights into compliance with lockdown measures and underscored the importance of implementing additional strategies to combat the pandemic.

### Pool testing: a pillar for the test, trace, and isolation strategy

Weeks before the World Health Organization declared the pandemic, Chile began preparing for one of the key pillars of its strategy: the molecular diagnosis of SARS-CoV-2 using RT-PCR. Led by the Chilean Public Health Institute (ISP), the testing capabilities in mid-February 2020 were not sufficient to provide fast and timely diagnoses to the entire population in the coming months. However, through resource injection, personnel hiring and training, and the commitment of health professionals, the number of laboratories authorized by ISP to carry out SARS-CoV-2 diagnosis progressively increased. By December 2020, 148 laboratories were implemented as part of the integrated SARS-CoV-2 diagnostic laboratory system, comprising public (44%), private (40%), and university (16%) sector laboratories, with a capacity of around 50,000 PCR reactions daily (18). This remarkable effort stands as one of the most significant achievements in managing the SARS-CoV-2 pandemic.

However, due to the high global demand for testing, one of the main difficulties was the availability of supplies and reagents for RT-PCR analyses, with shortages on the horizon. This situation prompted many laboratories to develop and implement new techniques or strategies to address supply availability and increase diagnostic capacity. These strategies included RT-PCR omitting viral RNA extraction (19), the use of in-home RNA extraction methods (20), MALDI-TOF techniques (21), and rapid antigen tests (22), among others. Among these strategies, pool testing emerged as a methodology with the greatest potential impact in Chile. Pool testing had already been used for other pathogens such as HIV, hepatitis B virus, hepatitis C virus, and influenza, including SARS-CoV-2 (23). Data generated at the Faculty of Medicine, University of Chile (6) and another national research group (24) suggested that pooling five samples was an appropriate approach in places where prevalence ranged between 3 and 15%. In such scenarios, the technique had the potential to increase testing capacity by 30 to 300%.

Recognizing the need to increase testing capacity, especially among the older adult population (arguably the most vulnerable segment), Chile’s National Service for the older adult (SENAMA) collaborated with ISCI and the University of Chile to develop a comprehensive strategy for conducting mass screening at long-term care facilities (LTCFs) (7). In May 2020, these institutions requested authorization from health authorities to pilot proactive, recurrent, and mass screening of residents and staff at two LTCFs. At that time, all testing efforts were conducted when infection was suspected, requiring individuals to remain in quarantine until test results became available, which could take several days. Due to the proactive nature of the proposed testing procedure, the health authority waived the quarantine requirements for the pilot. After obtaining support from the health authority and validating the pool testing technique locally, the population at these LTCFs was tested on a weekly basis for three consecutive weeks starting in early July 2020, using the pool testing technique. The logistical efforts, involving testing a population of over 100 patients, were conducted by the Asociación Chilena de Seguridad (ACHS), a nonprofit mutual insurance organization, and funded by the Confederación de Producción y Comercio (CPC), a private nonprofit business organization. By the end of the pilot, mass screening was successfully carried out, paving the way for the development of a testing strategy for SENAMA. Significantly, the pool testing approach required only 30% of the PCR reactions that would have been needed with individual testing (25).

Based on the pilot’s results, in September 2020, the health authority officially authorized a general framework for conducting pool testing in the clinical laboratory network and encouraged its use whenever positivity rates were around or less than 10% in the general population. Subsequently, most accredited laboratories incorporated pool testing as a fundamental pillar for the test, trace, and isolate strategy.

### Active search for asymptomatic infections: combining mobility and epidemiology

By the end of the first wave in September 2020, the PCR positivity rate among the general population had decreased to a level where reactive testing of symptomatic individuals and their close contacts required fewer tests than the available testing capacity. This, along with the implementation of pool testing techniques, liberated enough resources to begin proactively testing of the population, specifically targeting asymptomatic cases.

**Figure 2:**
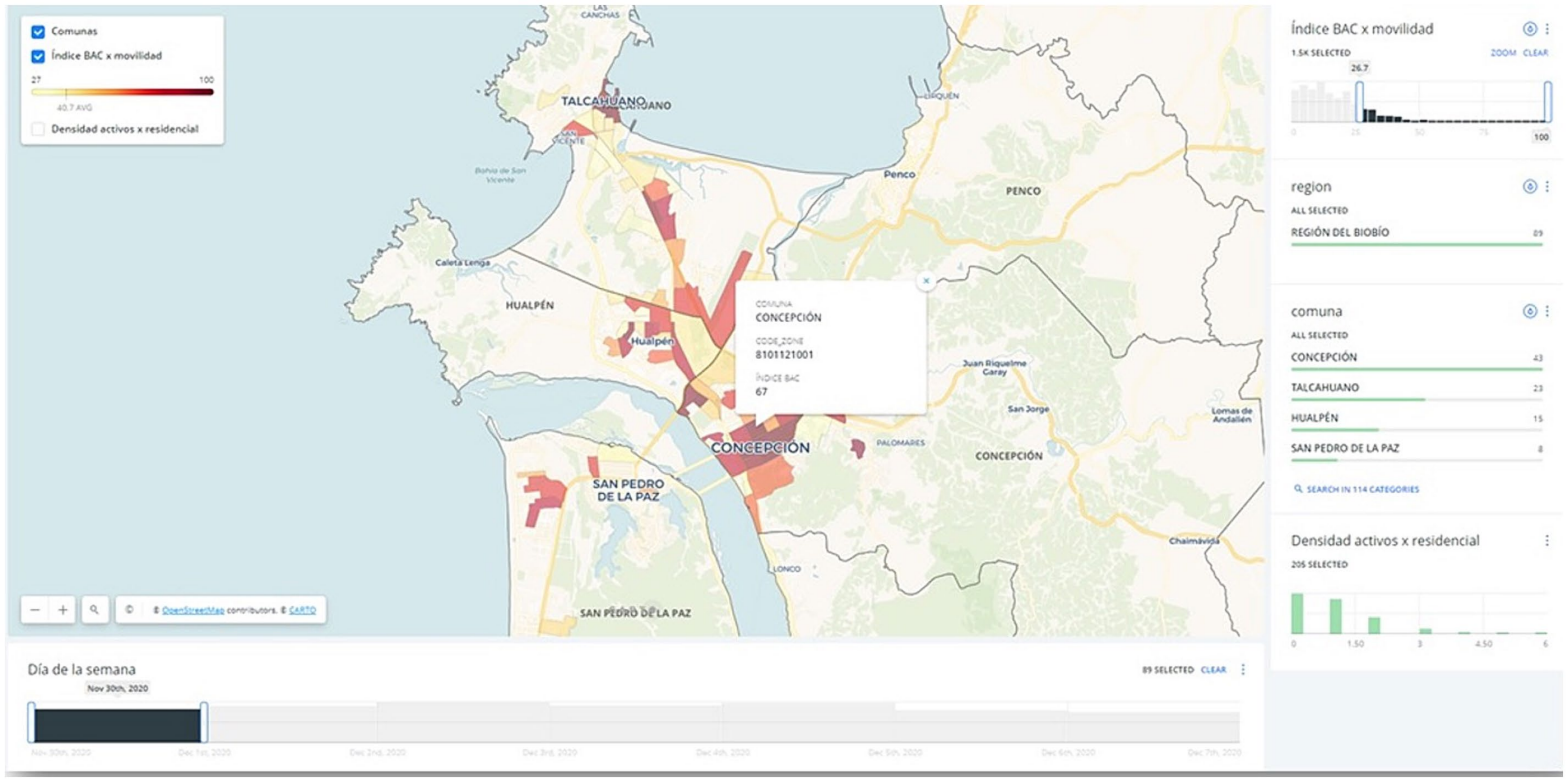
Graphic user interface of the BAC index, as seen by its users (showing the city of Concepcion): darker regions are census blocks in which there is higher estimated probability of detecting asymptomatic cases. Carto Maps, https://carto.com/.

Initially, local health authorities were responsible for directing testing efforts using spare capacity due to the unique nature of the situation. Concurrently, mobility data indicated that quarantines were ineffective after the first wave, as the population was largely commuting on a daily basis. Considering this, the local authority with jurisdiction over downtown Santiago, which had initiated active case searches by deploying mobile testing stations, reached out to ISCI for assistance in selecting locations to increase the detection of asymptomatic cases. ISCI combined real-time epidemiological data, which revealed the geographical distribution of new cases, with cell phone mobility data, which revealed daily population movement patterns, to predict the likelihood of positive testing outcomes at the census block level. This information was summarized in the form of an “active case search” (BAC in Spanish) index and communicated through a web platform using heat maps, see Figure 2.

Following the initial deployment of the BAC index in mid-October 2020, the national strategy for active search of asymptomatic cases in public spaces was formulated by the health authority, with the BAC index as its foundation. Computation of the index required up-to-date nationwide epidemiological information from the Department of Risk Management in Emergencies and Disasters of the health ministry, as well as granular mobility data from the telecom company Entel. Due to the large volume of data involved, the computation process took several days. Every Friday, massive raw mobility data was processed to generate forecasts of mobility patterns in each city. Then, on each Sunday, this information was combined with epidemiological data to produce the index before the active search planning meetings held at various organizations at the beginning of each week. As active search efforts played an increasingly important role in the national strategy, the heat map platform gained over a hundred users throughout the country by the end of 2021. This necessitated regular training sessions and addressing numerous customization requests to tailor the platform to the needs of local communities, ensuring its effective utilization. In practical terms, the information generated on a weekly basis supported decisions on future quarantines.

### Pilot testing of IgG seroprevalence using finger prick lateral flow anti-SARS-CoV-2 IgG antibody responses in downtown Santiago

In late 2020, during the early stages of collaboration with the health authority in the search for asymptomatic cases in public spaces, a significant focus was placed on analyzing the mobility patterns of people in downtown Santiago. This analysis was crucial in identifying the best locations for mobile testing facilities. Researchers from ISCI and the Faculty of Medicine at the University of Chile, who were actively collaborating at the time, recognized the opportunity presented by the high volume of people visiting certain downtown areas from all 50+ municipalities in the city. By setting up testing sites in strategic locations, for SARS-COV-2 antibody detection, they could test a representative sample of the entire city and gain insight into the state of population immunity.

With the experience gained from the first school outbreak and subsequent serological study, the researchers designed and conducted a pilot serological study among the population in the downtown area. This initiative was sponsored by the health authority in charge of downtown Santiago and recruited individuals through their active search program. Volunteers who provided informed consent underwent a finger prick for antibody testing using a lateral flow easy to read IgG antibody test. While waiting for their results, which took 15 min, volunteers completed a web-based questionnaire on demographics, health, and transportation information.

The collaboration of the Ministry of Science was crucial in making this initiative possible. They secured a donation of over 20 thousand IgG antibody tests from a large mining multinational company operating in the north of Chile. Although IgG antibody tests had lower precision compared to the gold standard PCR tests for individual diagnosis, they could accurately estimate the presence of IgG antibodies at the population level. After 2 months of operation in downtown Santiago, the collected information was used to create a representative sample of the city’s population. The results revealed, for example, that the estimated prevalence of the virus was nearly four times higher than the reported cases. The pilot results, which received significant media coverage (26) informed local discussions on the potential achievement of herd immunity in the future.

## Advancing in 2020: aggressive and early vaccination strategy

In May 2020, the Science Ministry presented its first confidential strategic report to the Presidency, recommending the evaluation of three emergency strategies. These strategies included local vaccine research and development (R&D) and manufacture, reconversion of plants for technology transfer and animal vaccine production, and the execution of international clinical trials to establish trust relationships with international companies for future vaccine supply benefits.

The focus was placed on the third alternative, and a specialized support team called the “Ministerial Scientific Advisory Committee for the Availability of a COVID-19 Vaccine” was quickly established (27) This committee consisted of top immunology, immunization, virology, and epidemiology specialists from universities, hospitals, and industry representatives across the country. Starting in July 2020, the committee began scouting and contacting potential vaccine candidates. The Science Ministry organized and directed meetings, while the committee members researched and reported on clinical trial ongoing results and provided formal recommendation dossiers. These dossiers included information on vaccine platforms, technical complexity, developer background, prior experiences, clinical research capacities at Chilean universities, and other relevant information to support clinical trials and future purchases. The committee also played a significant role in developing an accurate, trustworthy, and friendly public communication strategy.

Four laboratories, namely Sinovac, Janssen/Johnson & Johnson, Oxford/AstraZeneca, and CanSino, were selected for their clinical trials, which were conducted in various locations across the country.

The science minister (author AC was the former science minister), was also part of the President’s advisory board, which met weekly to assess the crisis and coordinate sanitary measures and emergency policies. On the President’s mandate, the science minister led the Inter-Ministerial Advisory Committee for the Availability of a COVID-19 Vaccine (28). This committee’s mission was to promote collaboration between local centers, universities, and international centers or laboratories dedicated to R&D. Its goal was to leverage Chile’s capabilities, advantages, and experience in conducting clinical trials to facilitate future vaccine access. The committee, headed by the Science Ministry, included representatives from the Ministries of Health and Foreign Affairs. It provided comprehensive advice to the Presidency based on each ministry’s expertise. The Science Ministry reported on scientific characteristics and clinical trial possibilities with the help of the Scientific Advisory Committee, while Foreign Affairs oversaw negotiations, and Health managed sanitary regulations and mass-vaccination strategies. Former Presidents and ambassadors in the United States, Europe, and China were actively involved in negotiations and dialogs with vaccine manufacturers.

On December 24th, 2020, Chile procured the first COVID-19 vaccine shipment (other than those required for clinical trials). 9,750 vaccines from Pfizer-BionTech were received by the President, along with the ministers of Foreign Affairs, Science and Interior. This shipment kick-started the national inoculation campaign, which prioritized 15 million people: emergency personnel, the older adult and chronically ill, and general population groups according to risk-exposure factors. The initial Pfizer doses were administered to intensive care unit workers in the most affected areas, Araucanía, BioBío, Magallanes and the Metropolitan Area.

The full vaccination campaign was officially launched on February 3, 2021. On June 23, 2021, the entire objective population had been successfully vaccinated with a full scheme (i.e., 15 million people). In December 2021, one year after receiving the first vaccine shipment, over 16 million had received a complete vaccination scheme, corresponding to 92% of the over-18 years old population. Additionally, 10 million people had received at least one booster shot.

Using a multi-platform strategic approach, with vaccines from Pfizer, Moderna, Jansen, AstraZeneca, Sinovac and Cansino, Chile ranked within the first two countries in COVID-19 vaccination coverage in the American region during the emergency (29). This represents a collective achievement based on a successful history of public health policies that includes public adherence, expert personnel and logistics.

### Tracking dynamic IgG seropositivity in Chile during vaccination

At the conclusion of the serology study pilot in February 2021, reports of the results drew the attention of health authorities responsible for coordinating public healthcare facilities across the country. Recognizing the potential for a large-scale nationwide serology study, the authorities proposed utilizing the same methodology as the pilot but at a low cost. Initially, the government had acquired a large number of IgG antibody tests early in the pandemic with the intention of using them to grant health passes that would exempt individuals testing IgG positive from constitutional exemption restrictions such as lockdowns. However, this idea was later abandoned due to limitations in the test’s operating parameters. Consequently, the health authority suggested utilizing these tests and funding over 30 testing stations throughout the country. The ISCI and the University of Chile agreed to provide the necessary technology for data collection and statistical analysis. In addition to the information collected in the pilot, questions regarding the vaccination status of the volunteers were added, considering the imminent vaccine rollout in the summer of 2021.

**Figure 3:**
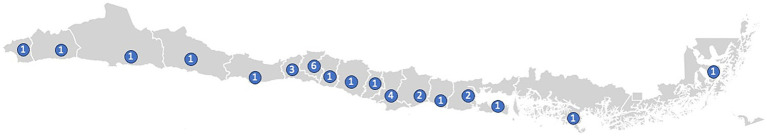
Location of the testing stations throughout Chile: 29 stations, one per national healthcare administrative unit, located through 24 major cities. Circles denote the number of testing stations in the area.

Starting in March 2021, testing stations were established in 24 major cities in Chile (Figure 3). Volunteers over 18 years old were invited to undergo a lateral flow test via finger prick and complete a web-based questionnaire. After a year of operation, the testing stations had gathered data from over 107,000 individuals.

One notable aspect of the study design was the use of mathematical programming techniques to select testing sites that would provide information representative of the population. The collected sample in this regard successfully replicated various characteristics of the general population.

An interim analysis conducted in early July 2021, prior to the decision on booster dose rollout, revealed crucial information. The data collected provided a clear overview of the vaccination rollout in the population, with the majority of individuals being unvaccinated at the study’s beginning. Subsequently, there was a growing proportion of individuals receiving first and second doses of the vaccine, predominantly the Sinovac inactivated virus vaccine, followed by a smaller proportion receiving the Pfizer vaccine. The rollout strategy followed a pattern of initially targeting the older adult population and progressively moving toward the younger population.

The analysis uncovered two key findings. Firstly, the vaccines were immunogenic, as there was a significant increase in IgG positivity among vaccinated individuals compared to the unvaccinated shortly after receiving the first or second doses. For those who received the Sinovac vaccine, the increase was moderate after the first dose and substantial after the second dose, reaching over 75% positivity. For Pfizer vaccine recipients, there was a significant increase after the first dose and a moderate increase after the second dose, with over 95% positivity.

Importantly, these results were in line with the countrywide effectiveness data for Sinovac generated by a research group based in the Health Ministry (8).

**Figure 4:**
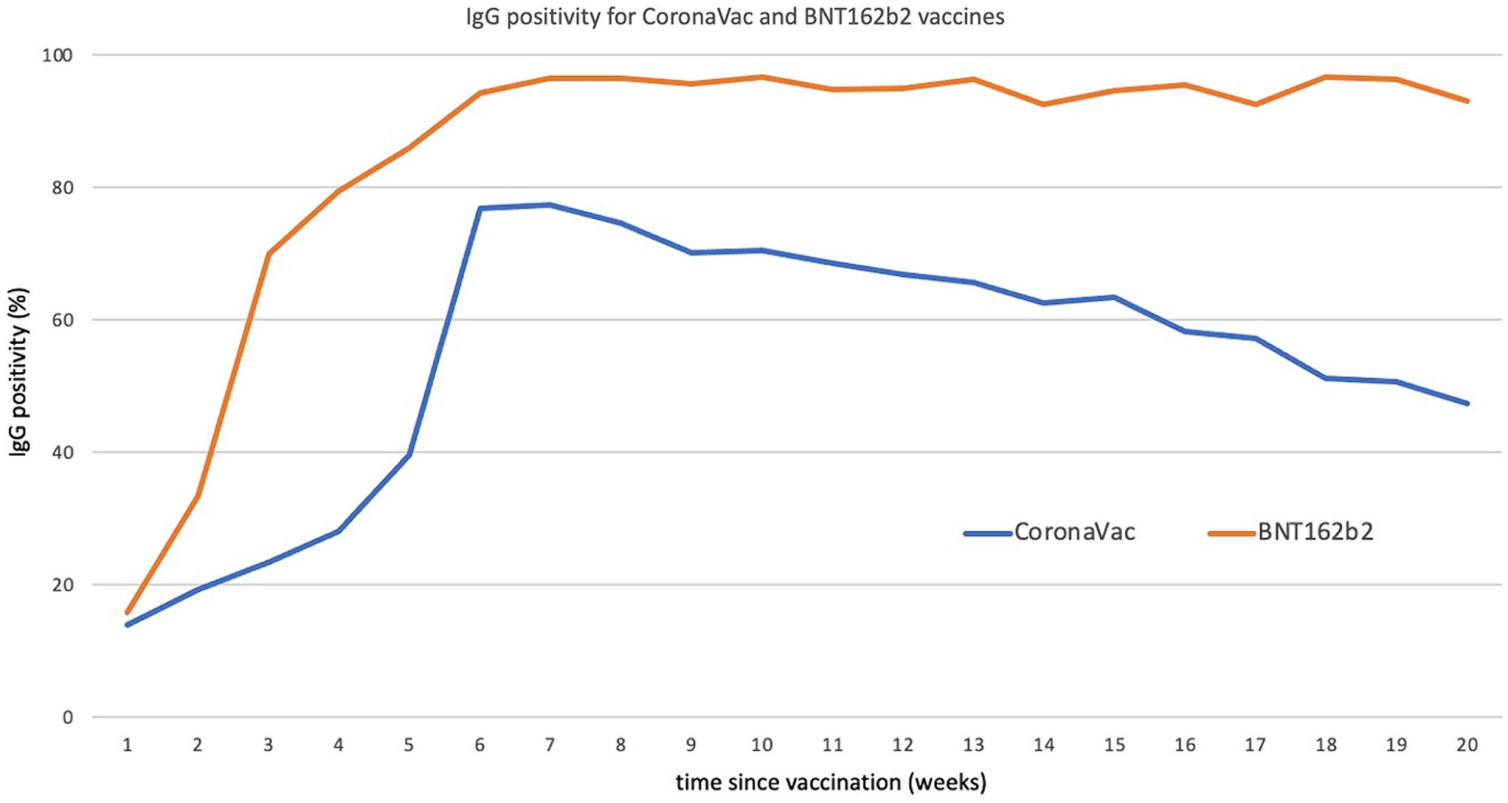
IgG positivity for CoronaVac and BNT162b2 as a function of time (in weeks) since vaccination. Source (9).

Secondly, while IgG positivity among recipients of the Pfizer vaccine remained steady at over 93% four months after the second dose, IgG positivity among Sinovac recipients steadily dropped to about 40% four months after, only slightly above the level of the unvaccinated population (Figure 4). This phenomenon was observed across genders and age groups, although it was more pronounced among the older adult. These results were published in late 2021 (9). They were shared with the health authority before, in early July, as a decision regarding the rollout of booster doses was expected at that time.

The rollout of booster doses began in August 2021, a few months before the FDA approved such measures in the US. For individuals who received the CoronaVac the inactivated vaccine from Sinovac primary vaccination, Chile implemented a strategy of heterogeneous booster vaccination. The most commonly used booster vaccine was Pfizer’s, primarily administered to individuals under 50 years old, followed by AstraZeneca/Oxford’s, predominantly given to those over 50 years old.

By the end of the national serology study, the analysis of the data confirmed the high effectiveness of this heterogeneous booster vaccination scheme. IgG positivity increased from approximately 40% to over 90% within 1 week of receiving the booster dose. (11). Subsequent epidemiological surveillance conducted by the health authority demonstrated substantial benefits of the heterogeneous booster dose scheme, including a reduced rate of COVID-19 cases, admissions to intensive care units, and deaths before the omicron surge.

### Gathering evidence on the correlation between lateral flow-based IgG and neutralizing antibodies

The national sentinel surveillance study focused on measuring immunity through the presence of IgG antibodies (referred to as IgG positivity), which raised some concerns. Generally, the presence of neutralizing antibodies is considered a more reliable indicator of the humoral immune response and a potential correlate of protection. To address this, researchers from ISCI and the University of Chile conducted a study to gather evidence on the correlation between IgG and neutralizing antibodies. The aim was to infer a measure of protection based on the results of the surveillance study.

**Figure 5:**
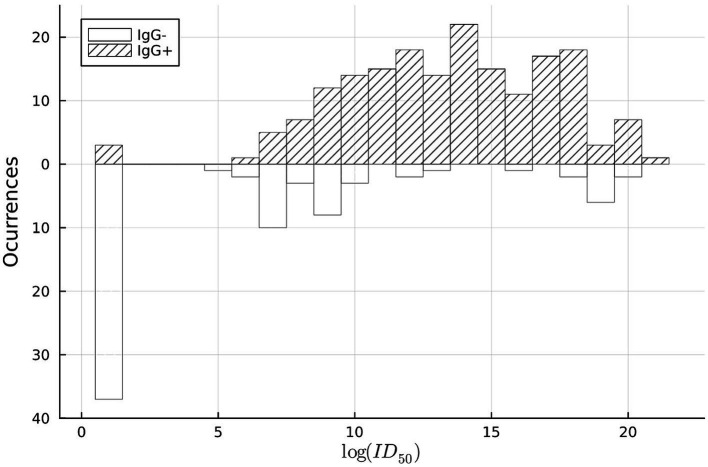
Histogram comparing COVID-19 IgG positivity to neutralizing antibody titer count, for the onsite lateral flow test. Source (11).

Between August 2021 and April 2022, volunteers over 18 years old seeking medical care at the Mutual de Seguridad de la Cámara Chilena de la Construcción, a non-profit mutual insurance organization, provided blood samples for IgG and neutralizing antibody studies. Our findings revealed a moderate correlation between IgG positivity and the log of the infectious dose in 50% of neutralizing antibodies (Figure 5). The sensitivity and specificity of the IgG lateral flow test, determined by comparing the results with plaque reduction neutralization, were close to those specified by the manufacturer.

Therefore, based on these results, it could be concluded that monitoring population IgG seroprevalence is a feasible and plausible way to infer changes in protection within the population until more affordable and easily implementable tests for neutralizing antibodies become available (11).

### Tracking dynamic IgG seropositivity in Chilean children

Chile implemented a national childhood vaccination campaign at a rapid pace, with approximately 80% of children aged 3–17 years fully vaccinated by February 2022. Starting from June 22, 2021, children aged 12–17 years received the mRNA Pfizer/BioNTech vaccine, followed by children aged 6–11 years who received the inactivated Sinovac vaccine. This required generating evidence quickly regarding the immune response to COVID-19 vaccines in the pediatric population, as there was limited information on whether the response was similar to or different from that in adults. It was also important to understand if there were differences in the response to different types of vaccines (inactivated versus mRNA) and how this response was maintained over time. It is worth noting that Chile and Latin America experienced one of the longest school closures globally, which had negative effects on children’s education and their mental and physical health.

We conducted a study involving 2,302 vaccinated and non-vaccinated Chilean school-aged children in the three main regions of the country who received the Sinovac inactivated vaccine (CoronaVac), the Pfizer/BioNTech mRNA vaccine (BNT162b2), or no vaccine within 1–20 weeks prior to sample collection. Two weeks after the second dose of the inactivated vaccine (Sinovac), more than 90% of the children demonstrated IgG seropositivity, which remained high for up to 10 weeks after the second dose followed by a steady decline. For the mRNA vaccine (Pfizer/BioNTech) this decline was not observed (12). Compared to the adult population (9), children showed a slightly weaker response to the mRNA vaccine and a slightly stronger response to the inactivated vaccine in terms of the overall proportion of individuals with seropositivity in the short term after vaccination. However, similar to adults, seropositivity for the inactivated vaccine declined over time, indicating a potential need for a booster dose in children.

Vaccinating children against COVID-19, ideally starting from an early age (6 months old), not only reduced the risk of infection and severe illness caused by SARS-CoV-2 but also helped mitigate the significant social impact that the pandemic has had on children. Unfortunately, this was not followed by desirable school reopening due mainly to opposition from the educational community.

## Main lessons and future perspectives

The experience gained during the COVID-19 pandemic has taught us several valuable lessons. One important lesson is the significance of positive political leadership, irrespective of political affiliations, that is open to input from the academic and scientific community. In Chile, this was exemplified by the role of various governmental entities in effectively managing the crisis.

Another crucial aspect was the rapid response and commitment of scientists and researchers from diverse disciplines. Their expert approaches and contributions to open data sets generated by government scientific authorities were essential in addressing the pandemic.

Transdisciplinary approaches, combined with proactive collaboration between academic, private, and governmental institutions, played a key role in finding effective solutions to the multiple problems posed by the pandemic. This required open-mindedness and generosity from all parties involved.

In addition, it became evident that identifying the right questions and providing evidence to address urgent public health problems should take priority over purely academic interests. This shift in focus allowed for better decision-making and policy responses.

According to highly conservative estimates, the number of lives saved by all the initiatives combined is close to 3,000, equivalent to more than 5% of the total death toll in Chile associated with the pandemic until January 2022. The saved resources associated with testing, ICU beds, and working days amount to more than 300 million USD. Details of this imjpact can be found in the following reference (30).

The successful collaborations between the Chilean Health Ministry and academic institutions have paved the way for applying the gained experience to other relevant health topics. For instance, the mathematical models used to identify ICU bed requirements during the pandemic are now being adapted for predicting bed requirements during the annual winter virus epidemic in Chile. These models, applicable to different regions of the country, will greatly aid in health planning for the future.

Furthermore, the trust and confidence established between the government and academic institutions have facilitated data sharing from various national databases. The ISCI is conducting advanced analyses on this data, including projections of the impact of respiratory syncytial virus on hospital admissions during winter epidemics. Additionally, ISCI and the Faculty of Medicine, University of Chile are assessing and modeling the potential impact and cost-effectiveness of novel preventive strategies, such as RSV monoclonal antibodies followed by effectiveness data after national implementation of these preventive strategies.

Overall, the lessons learned from the COVID-19 experience in Chile have highlighted the importance of political leadership, collaboration, and evidence-based decision-making in effectively addressing a major public health crisis.

## Author contributions

JT: Conceptualization, Writing – original draft, Writing – review & editing. LB: Conceptualization, Writing – original draft, Writing – review & editing. DS: Conceptualization, Writing – original draft, Writing – review & editing. MZ: Writing – original draft. MF: Conceptualization and Writing – original draft. VM: Writing – original draft. NC: Writing – original draft. MO’R: Conceptualization, Writing – original draft, Writing – review & editing. AC: Conceptualization and Writing – original draft.

## Footnotes

^1^ Sadly deceased February 6, 2024.
